# Point Score Systems and Cooperative Incentives: The 3-1-0 Curse

**DOI:** 10.3390/sports6040110

**Published:** 2018-09-30

**Authors:** Kjetil K. Haugen, Knut P. Heen

**Affiliations:** 1 Faculty of Logistics, Molde University College, Box 2110, 6402 Molde, Norway; 2 Faculty of Business Administration and Social Sciences, Molde University College, Box 2110, 6402 Molde, Norway; knut.p.heen@himolde.no

**Keywords:** game theory, point score system, adverse incentives, empirical analysis, Z280, Z200, L51

## Abstract

The purpose of this study is to analyze the consequences that point score systems in association football may have on potential collusion between teams. The study applies game theory and empirical analysis to derive and test hypotheses. The main findings of the article include Nash equilibria indicating a higher collusion potential associated with the 3-1-0 point score system than with the 2-1-0 system. Of particular interest is the finding that the competitive balance of the league affects collusion, and that (theoretically) high competitive balance in fact makes collusion more probable. Empirically, we are not able to prove that real-world participants do collude, but we provide circumstantial evidence that is consistent with collusion. The empirical analysis is based on a sample of 25 European top leagues with 823 played matches in the 2017 and 2016/17 seasons. These data are used to estimate uncertainty of outcome and draw ratio. We apply a standard *t*-test to test our main hypothesis. The main conclusion of the paper may hence be summed up as advice to reinstall the 2-1-0 point score system in association football.

## 1. Introduction

Scoring systems in sports often inspire debate. Did the three-points rule make English football great again? There are many opinions. Jimmy Hill, the father of the rule, claims the rule “revolutionized” football. Former Arsenal manager Terry Neill gave the rule a lukewarm welcome when he predicted that teams leading by one goal would play more defensively [1].

The Football League first introduced the 3-1-0 system in England in 1981 [2], thereby replacing the original 2-1-0 system. The original 2-1-0 system was an evolution from the older money-prize-pot system associated with the challenge matches that pre-dated the foundation of the Football League in 1888 [1]. The prize pot system was simply a winner-takes-all system that allowed for splitting the pot if there was a draw. The fact that there was a prize of a given size meant that the prize pot system had to be a zero-sum game for budget reasons (It is a zero-sum game once the game starts. They split the pot if there is a draw. The winner gains what the losers lose if one team wins). The modern 3-1-0 system is not a zero-sum game because the winner gains more points than the loser loses. This is possible because points are not subject to a budget constraint like a monetary prize. The difference between a zero-sum game and a non-zero-sum game is that there is nothing to gain by cooperation in a zero-sum game. In a non-zero-sum game, however, there may be something to gain by cooperation although that outcome may be difficult to achieve, like for instance in the Prisoner’s Dilemma. The fact that the 3-1-0 system may lead to cooperation should perhaps have raised some eyebrows in 1981.

The difference between the two systems is that winning matches is more attractive in the 3-1-0 system than in the 2-1-0 system. In the 2-1-0 system, one win and one loss give the same point score as two draws, 2 points. In the 3-1-0 system, one win and one loss give more points than two draws, 3 points versus 2 points. The intention behind the 3-1-0 system is to provide stronger incentives for the participants to take risk, i.e., try to win instead of settling for a draw. The Football League introduced the system because attendances had been falling since the 1950s. They cited so-called dull play as their prime reason [1]. Likewise, FIFA (Fédération Internationale de Football Association) introduced the 3-1-0 system before the World Cup in 1994 because they were concerned about how a US audience would deal with draws [1]. All the important leagues also adopted the 3-1-0 system during the same period [2].

The problem with the 3-1-0 system is that it provides incentives to cooperate because it is not a zero-sum game. The winner gains more than the loser lose as compared to a draw. The 3-1-0 system is therefore also more suited for match fixing than the 2-1-0 system. Obviously, the rules forbid outright bribes. There are however many close substitutes to monetary bribes that are very difficult to detect and to prove. In most football leagues each team meet each other twice a season, home and away. Two teams who fear two draws may therefore agree to one win each instead. Both teams will then receive 3 points instead of 2 points. Such incentives are completely absent in the 2-1-0 system because both alternatives give 2 points. The incentive to cooperate is weaker if one team is significantly better than the other team. In the present paper, we show exactly how large the quality difference must be for the collusion solution to fall apart. The problem with the 3-1-0 system is that the incentive to fix matches is at its greatest when the teams are of exact equal quality. We therefore end up with the result that there are two types of matches in the 3-1-0 system. Type one is competitive matches in which one team is so much better than the other team that we already “know” the result in advance. Type two is matches between teams of similar quality that may be subject to match fixing.

So far, we have treated teams as exogenously given. In modern football, however, trading of players is an important part of club football. The point-scoring system also affects the incentive to trade players. Suppose we start out in a situation in which the probability distribution of each individual game is a certain draw. We may define such a league as a perfectly balanced league (We define a perfectly balanced league as a league in which all teams face the probability of 1 for a draw). Suppose as an example that two teams trade a player such that the buying team’s probability distribution changes to (W: 0.01; D: 0.99; L: 0.00) and the selling team’s probability distribution changes to (W: 0.00; D: 0.99; L: 0.01). Under the 2-1-0 system, the buying team will expect to get 1.01 points per game while the selling team will expect to get 0.99 points per game. Trading the player is a zero-sum game here. Under the 3-1-0 system, the buying team will expect to get 1.02 points per game while the selling team still expect to get 0.99 points per game. Trading the player is no longer a zero-sum game. The trade has created 0.01 points out of nowhere. In other words, the 3-1-0 system allows teams to create points by buying players. A perfectly balanced league is therefore not a Pareto efficient equilibrium under the 3-1-0 system. Indeed, there is an incentive to get rid of all draws via trades. Any equilibrium without any draws is Pareto efficient under the 3-1-0 system, including a perfectly imbalanced league (We define a perfectly imbalanced league as a league in which the best team wins all its matches; the worst team loses all its matches; the rest win all its matches against teams below them on the table), (A perfectly imbalanced league is Pareto efficient because you cannot change any of the results such that one team gets more points without reducing any other team’s points score. A more balanced league with many potential draws is not Pareto efficient because any team would be willing to trade two draws for a win and a loss). In a zero-sum game, as in the 2-1-0 system, all equilibria are Pareto efficient. This does not guarantee a perfectly balanced league, but the 2-1-0 system is at least not hostile towards a perfectly balanced league.

The Football League introduced the 3-1-0 system because they feared that the league was heading towards a situation in which dull draws became the rule rather than the exception. Mathematically speaking, we may say that they feared the league was moving towards the following distribution (W: 0.0; D: 1.0; L: 0.0). The intention was that the 3-1-0 system would remove some draws through more attacking (risky) play without skewing the distribution. They were in other words hoping for a move in the direction of the following distribution (W: 0.5; D: 0.0; L: 0.5). The problem is that the 3-1-0 system penalizes draws. It does not promote attacking play per se. We should not expect more attacking play if there are alternative means to achieve the end (fewer draws) that are more suitable. After all, if teams are of similar quality, attacking play may produce exciting 5-5 draws too. The 3-1-0 system does not reward attacking play. It penalizes draws. The simplest way to get rid of draws in the current system may be either through outright match-fixing schemes, or through changing the quality composition of the league such that no two teams are of equal quality.

What was the result of the change? From 1946 to 1981, 27 (These draw ratios are calculated by data gathered from [3]) percent of matches in the English top division ended in a draw. After 1981, 27 percent of the matches ended in a draw. The only major difference between the two periods is that the top five teams each season have won significantly more away matches during the latter period than the former period, 44 percent vs. 37 percent. Moreover, Haugen and Heen [4] document that there has been a dramatic fall in competitive balance in the English top division from the mid-1970s onwards.

Our article contributes to the growing literature on potential adverse effects of the 3-1-0 point score system in sports. The 3-1-0 literature is however just one branch of a much larger literature on the unintended consequences of human choices. Although unintended consequences may be both positive and negative, most of the literature focuses on well-meaning choices that comes with bad side effects. Adam Smith’s invisible hand is a noteworthy exception (Wealth of Nations: “*It is not from the benevolence of the butcher, the brewer, or the baker that we expect our dinner, but from their regard to their own interest*”). Our article is perhaps closest in spirit to Kerr [5]. He warns us of the folly of rewarding A while hoping for B. The 3-1-0 system penalizes draws (compared to the 2-1-0 system) while the supporters of the 3-1-0 system is hoping for more attacking football. Garciano and Palacios-Huerta [6] investigate a related question to ours. They argue that the 3-1-0 system provides stronger incentives for holding onto a lead than the 2-1-0 system, and thus provides stronger incentives to sabotage the opponent’s attempts to score goals. They also provide evidence, which they interpret in favour of their sabotage hypothesis.

An important contribution, directly related to the problem we discuss here, was made by Shepotylo [7]. Here, the author applies game theory to analyse a team’s incentives under various point-score systems, and concludes—after an empirical analysis—that the 3-1-0 system had mixed results. He claims that the new system had positive effects (Quote:“*more attacking style in play as well as more scored goals*”) in competitive leagues. However, in less-competitive leagues, he concludes that it has had a negligible effect on scoring, and even has led to increased corruption. Our results will indicate a different story. We show that the more competitive the league is, the more likely collusive behaviour (match fixing or point sharing) will be.

Other potential adverse effects of the 3-1-0 system has been discussed broadly. Haugen [8,9,10], has for instance discussed various potential adverse effects of the 3-1-0 system ranging from team to coach incentives. A suggestion of changing the point score system in an even more radical way as proposed by Fernandez-Cantelli and Meeden [11], and later criticised in [12]. Brochas and Carillo [13] discuss effects of point score systems, mainly focusing on the (later abolished) “*golden-goal rule*”, while Hon and Parinduri [14] perform an empirical analysis of German Bundesliga, concluding that they do not find evidence that the three-point rule makes games more decisive, increases the number of goals, or decreases goal-differences. However, Moshcini [15] claims the opposite in a paper published 4 years earlier than Hon and Panduri. It seems safe to conclude that research provides conflicting views on the consequences of the introduction of the 3-1-0 system.

The main objective of this paper is to demonstrate, applying game theory modelling, that the 3-1-0 point score system in football introduced extended adverse incentives for collusion compared to the previous 2-1-0 point score system. We apply two different gaming techniques to arrive at our main result. Furthermore, this hypothesis is tested empirically. Proving collusion (empirically) beyond reasonable doubt is hard. Proving collusion beyond scientific doubt is even harder. We base our empirical strategy on falsification [16] rather than verification [17]. We base our null hypothesis on the idea that teams of equal quality will draw more often than teams of unequal quality (if collusion is absent). We should therefore find that a balanced league produces a higher draw ratio than an imbalanced league. We test the hypothesis on a sample of 25 European leagues for the 2017 season. We find no relationship between the competitive balance of the league and the draw ratio of the league. The finding does not prove collusion, but it falsifies the idea that teams of equal quality share the points “naively” in the form of draws.

## 2. A Simple Game Model

We start with a very simplified game model. There are two (perfectly) equally good football teams ($T_{1},T_{2}$) playing two matches (The two teams are presumably engaged in a double round robin tournament) against each other. These two matches allow each team to choose from two possible actions (strategies):

*N*: Play normally
*A*: Make an agreement sharing two victories—a home win for each

We assume further that both teams maximise expected point score from the given general point score system (W,D and L denote a win, a draw or a loss respectively. The assumption ω>δ>0 is evident):

W: ⇒ ω points
D: ⇒ δ points
L: ⇒ 0 points

To make things as simple as possible, we assume that both teams will have to stick to their action throughout both matches. Then, given that we introduce a simple simultaneous imperfect information game, and an underlying assumption of maximal expected point score as the teams’ objectives, the following pay-offs seem obvious.

*A*,*A* If both teams choose the *A*-action, they share the total amount of points after one win each. Hence, ω to both players.
*N*,*N* Without any agreement (choosing *N*), both teams plays as usual, and by being perfect clones, will have a probability of $\frac{1}{3}$ for each outcome W,D and L. Hence, $2\cdot \frac{1}{3}\left(\omega +\delta \right)$ to both players (Recall 2 games are played).
*A*,*N* (or *N*,*A*). Now, our model is a bit too simplified. We overlook the obvious inherent sequentiality here; one team may observe that the other deviates, and hence punish back. Furthermore, there may very well be some effects of incomplete information as well, making it even more complex. Anyway, what we can establish is that the remaining pay-offs must be located within the following interval:
  (1) $$\left[\min\left\{\omega ,\frac{2}{3}\left(\omega +\delta \right)\right\},\max\left\{\omega ,\frac{2}{3}\left(\omega +\delta \right)\right\}\right]$$

Now, the interesting thing here happens if:

(2) $$\omega =\frac{2}{3}\left(\omega +\delta \right)\Rightarrow 3\omega =2\omega +2\delta$$

or

(3) $$\begin{array}{c} \omega =2\delta \end{array}$$

Refer to Appendix A for an alternative, and in some ways simpler method, to derive a similar result.

**Figure 1:**
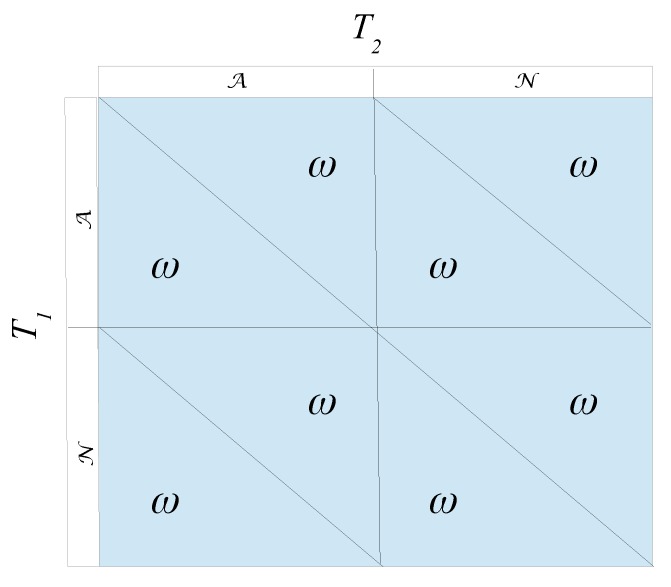
The “game” which is really not a game.

In this case, the interval (Equation (1)), collapses to

(4) $$\left[\min\left\{\omega ,\frac{2}{3}\left(\omega +\delta \right)\right\},\max\left\{\omega ,\frac{2}{3}\left(\omega +\delta \right)\right\}\right]=\left[\min\left\{\omega ,\omega \right\},\max\left\{\omega ,\omega \right\}\right]=\left[\omega ,\omega \right]=\omega ,$$

and the resulting “game” as shown in Figure 1:

Obviously, a game which provides the same pay-off whatever strategies are chosen is not a real game. It does not matter what the players do. Point score systems which produce this outcome are the old 2-1-0 system in football alongside the present system in chess; 1, $\frac{1}{2}$, 0, to name two examples. The 3-1-0 (present point score system in football) does **not** collapse to a non-gaming situation.

What happens here is evident. A point score system where ω=2δ provides no incentives for sharing a win between the two teams, as sharing a win (agreeing to let the home team win for instance) provides two points to each team. Playing normally, and utilizing the assumption of perfect team quality clones, the expected outcome to each team is also 2 points, two draws. Hence, there are no incentives (weakly) to go for an “agreement solution”. And, both teams may as well play normally. That is, there are no incentive problems. In a situation where ω≠2δ, for instance in the 3-1-0 system (3≠2·1), things are substantially different. Now, an agreement of sharing two home victories makes economic sense. In such a situation each team could achieve 3 points each, as opposed to the “playing normally” situation, where they (on average) would achieve only 2 points each—after two draws.

Hence, the 3-1-0 point score system has a fundamental flaw. It allows for agreements between teams, agreements that may affect strategies and performance. Even though we have limited suspicion of teams actually making these types of agreements, tacit collusive behaviour seems possible. This means that, say, two top teams, more or less equally good, may very well (without making any formal binding contracts) end up cooperating along a similar agreement-strategy. This is obviously not good for football.

**Figure 2:**
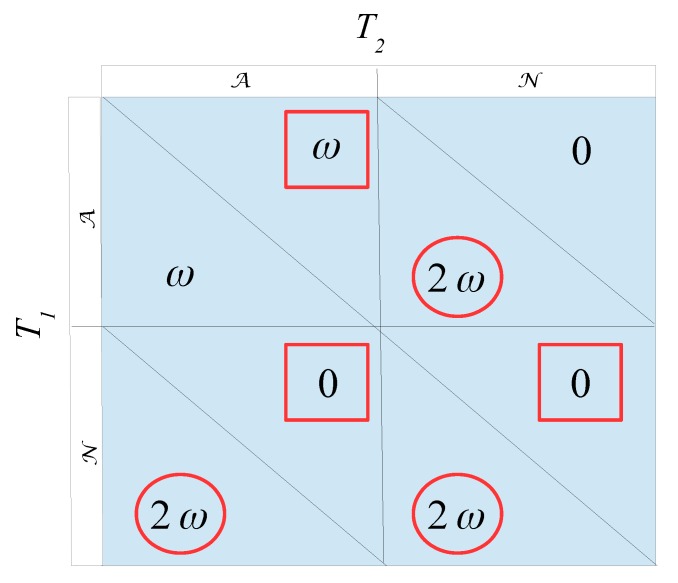
The game with two maximally uneven teams (quality-wise).

So, what happens if the two teams are not perfect clones? That is, one team is better than the other, say (without loss of generality) $T_{1}$ is better than $T_{2}$. It may prove simpler to investigate the extreme case first. That is, $T_{1}$ is perfectly better than $T_{2}$. In such a situation the initial “clone” assumption changes the original probability distribution from $\left[\frac{1}{3},\frac{1}{3},\frac{1}{3}\right]$ to $\left[1,0,0\right]$, and $\frac{2}{3}\left(\omega +\delta \right)$ changes to 2ω. The resulting game changes accordingly, as shown in Figure 2—red circles and squares indicate best replies for $T_{1},T_{2}$:

The game in Figure 2 predicts the obvious. When $T_{1}$ always beats $T_{2}$, $T_{1}$ will always play normally (choose the N-strategy in Nash equilibrium (NE)). It does not matter what $T_{2}$ does, either choosing the A or N-strategies, or any probabilistic combination of them. Hence, this game contains 2 pure-strategy NEs—{N,A} and {N,N}—as well as an infinite amount of mixed-strategy NEs, where $T_{2}$ can choose any mixing probability in [0,1]. Or, maybe simpler, in plain English: If $T_{1}$ always beats $T_{2}$, $T_{1}$ will always play normally (to win), and achieve 2ω points in any double match between the two teams.

In order to enhance this argument, we need to introduce parametric probabilities for various match outcomes. We define:

$$\begin{array}{ccc} p_{1} & : & \mathrm{Pr}(T_{1}\;\mathrm{beats}\;T_{2})\\ p_{2} & : & \mathrm{Pr}(T_{2}\;\mathrm{beats}\;T_{1})\\ P_{D}=1-p_{1}-p_{2} & : & \mathrm{Pr}(\;\mathrm{Draw}\;) \end{array}$$

Now, It ought to be likewise clear, that at some point in between the two probability distributions, $\left[\frac{1}{3},\frac{1}{3},\frac{1}{3}\right]$ and $\left[1,0,0\right]$, there is a flip between the two potential NEs–{A,A} and {N,N}. Logically, this must happen when

(5) $$2\left[p_{1}\omega +\left(1-p_{1}-p_{2}\right)\delta \right]>w\Rightarrow 2\left(1-p_{1}-p_{2}\right)\delta >\omega \left(1-2p_{1}\right).$$

Now, $1-p_{1}-p_{2}>0$ (A non-zero $p_{D}$ seems reasonable). Then, Equation (5) can be written:

(6) $$\frac{\delta }{\omega }>\frac{1-2p_{1}}{2(1-p_{1}-p_{2})}.$$

Some slight algebraic manipulations of Equation (6) give:

(7) $$\frac{\delta }{\omega }>\frac{1}{1+\frac{1-2p_{2}}{1-2p_{1}}}.$$

This relation is interesting, as it “binds” together the choice of point-score system ($\frac{\delta }{\omega }$) with the quality difference ratio of the teams ($\frac{1-2p_{2}}{1-2p_{1}}$). Again, the magic of the 2-1-0 system appears, as our previous assumptions lead to $p_{1}\geq p_{2}\Rightarrow \frac{1-2p_{2}}{1-2p_{1}}\geq 1$ or

(8) $$\frac{\delta }{\omega }>\frac{1}{2+\epsilon }$$

where $\epsilon ,\left(\epsilon \geq 0\right)=\frac{1-p_{2}}{1-p_{1}}-1$.

Let us, as a final remark, revert to the game in Figure 2. One obvious feature of the NEs is that there is no incentive problem any more. $T_{1}$ plays normally in equilibrium, while $T_{2}$ can randomize freely between A or N. Assuming some cost involved in acting slightly outside the standards of fair play, i.e., choosing A, it seems reasonable to assume that also $T_{2}$ will choose N. Consequently, in a situation (for any given point score system) where $T_{1}$ is sufficiently better than $T_{2}$—inequality (7) is satisfied—the incentive problem vanishes. So, if all teams are strictly ordered in a league, the best team (almost always) beats the second best, the second best (almost always) beats the third best and so on, and there will be no incentive problems with any point score system (as long as ω>δ>0. However, it might be that such a league is so imbalanced that the audience has lost interest a long time ago. Getting rid of the incentive problem does not really help, if you get rid of the audience at the same time.

## 3. Theoretical Discussion and Conclusions

Apart from the obvious conclusion that point score systems where ω≠2δ should be used with great care, our inequality (7) is perhaps our most interesting finding. As discussed briefly in the introduction, it gives us the exact quality difference between teams needed for the collusion solution to fall apart. For instance, entering the 3-1-0 system into inequality (7) (δ=1 and ω=3) provides an expression where quality differences (by $p_{1}$ and $p_{2}$) can be entered to judge “where these two teams are” with respect to potential collusive behaviour. As such, it could provide relevant decision support.

The fact that our model predicts more potential for collusive behaviour the more balanced the league is is interesting. This allows for alternative empirical strategies, for instance where uncertainty of outcome can be used as one of the variables.

## 4. Empirical Analysis

Our theoretical analysis predicts that there will be more collusion under a 3-1-0 system than under a 2-1-0 system. The reason is that there is no incentive to collude in a 2-1-0 system (except under special circumstances towards the end of the league). Ideally, we would run an experiment in which we play a league with a 2-1-0 system and record the collusion in the league, then we would re-run the very same league (holding everything else constant) with a 3-1-0 system and record the collusion in the league, and finally we would compare the collusion in the two runs with each other. It is difficult to run the experiment for many reasons. First, collusion is an unobservable variable. Second, you cannot re-run the very same league with different point scoring systems. It is therefore tough to test our prediction using the scientific method.

Our empirical strategy is to mimic the ideal experiment as closely as possible. Today, all major football leagues use the 3-1-0 system. This leaves us with two possible approaches. Either we may compare the 3-1-0 system period with the 2-1-0 system period, or we may compare today’s different leagues with each other. The latter is possible because our theoretical analysis predicts that the incentive to collude is weaker in imbalanced leagues than in balanced leagues (because quality differences between teams prevent collusion). We choose the latter approach because very little remains constant over time. For example, the amount of money involved has changed considerably since the introduction of the 3-1-0 system. There is simply more at stake today than there used to be.

Collusion is an unobservable variable. The consequence of collusion is however observable. Teams, which expect to play two draws during a season, would gain one point each if they decide to share two wins instead. An unusually low draw ratio in a league may therefore be a symptom of collusion. The draw-ratio of a league is also directly dependent on how balanced the league is. In the absence of collusion, we expect to see a higher draw-ratio in a balanced league than in an imbalanced league. Under the presence of collusion, however, we expect no particular relationship between the draw ratio and the balance of the league. The point is that a more-balanced league should produce a higher draw ratio (absent collusion), but because the incentive to collude works in the opposite direction, we may even observe a lower draw ratio in a balanced league than in an imbalanced league (if collusion is present). Our approach is similar to Duggan and Levitt [18] who, instead of searching for evidence of collusion, search for evidence inconsistent with “honest” competition.

Our sample consists of most European leagues for the season ending in 2017 (See Table 1). Some countries, such as Belgium, Denmark, and Scotland, use a play-off system to determine the winner. We drop these leagues from our sample. For each league, we compute the draw ratio, δ, and our uncertainty of outcome measure, $\rho_{L}$ (See Appendix B.2). The draw-ratio is simply the number of draws during the season divided by the number of games during the season. The uncertainty of outcome measure is a percentage measure that produces 100 percent if the league is perfectly balanced, and zero percent if the league is perfectly imbalanced.

Table 1 presents the relevant measures for each country. We see that uncertainty of outcome varies quite a bit between the most imbalanced league (10.1 percent, Croatia) and the most balanced league (40.3 percent, Hungary). The draw-ratio varies between 18.9 percent in Austria and 32.8 percent in Albania.

**Figure 3:**
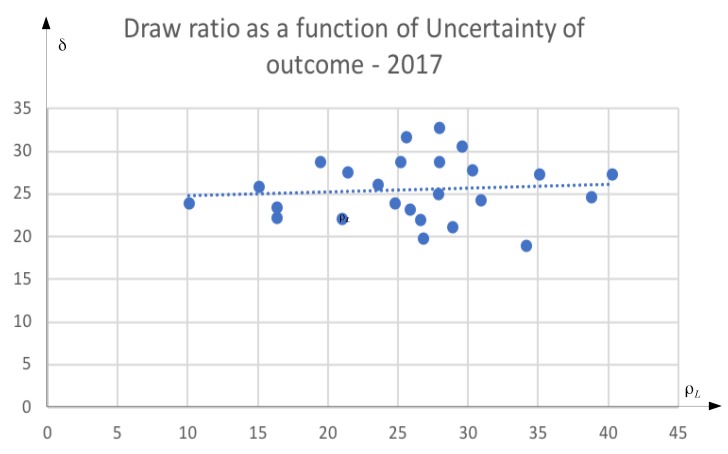
Plot of $\delta_{j}$ and $\rho_{l}^{j}$ with estimated regression line.

Figure 3 presents the data in the form of a scatter plot with uncertainty of outcome on the *x*-axis and the draw-ratio on the *y*-axis. We see that the draw-ratio is symmetrically distributed around a mean of 25 percent, and that the draw-ratio is independent of how balanced the league is. We fit an ordinary least-squares regression to the data, and report the results in Table 2.

The regression coefficient on the uncertainty of outcome variable is close to zero and statistically not different from zero. The regression line is flat. Our interpretation is that the data is inconsistent with “honest” competition.

We have identified an empirical puzzle. Probability theory tells us that there should be a higher draw ratio in leagues hosting teams of similar quality, but the data tell us otherwise. Why? Our theoretical model provides one explanation consistent with the data. Teams of similar quality have a stronger incentive to share two wins instead of risking two draws than teams of different quality. We cannot prove that collusion is the only explanation, but we have at least falsified the idea that the competitive balance of the league determines the draw ratio of a league. The latter is a quite surprising finding if teams actually compete honestly.

## 5. Discussion, Conclusions and Suggestions for Further Research

We have argued that the 3-1-0 point scoring system creates an incentive for teams to collude. It is better to win one match and lose one match than to draw two matches. This incentive is absent in the 2-1-0 system. We have shown that the incentive is more relevant in leagues consisting of teams of similar quality, i.e., more competitive leagues. We base our empirical analysis on a sample of 25 European top leagues with 823 matches played during the season ending in 2017. Basic probability theory tells us that the draw ratio should be higher in the competitive leagues. Our statistical test, however, cannot reject the hypothesis that the draw ratio is identical, 24 percent, across all leagues. This does not prove the existence of collusion, but it is certainly suspicious.

We feel that these results should be of interest, and hopefully stimulate to further research. As pointed out in the introduction, actual research into this area is sparse. As far as we have been able to identify, only contribution [7] exists presently, and after all, as point score systems are relatively easy to change with an affordable cost, why not consider either changing back, or investigate other possibilities? Hence, more research is necessary. Surely more advanced empirical studies than ours, involving time and/or more direct measurements of club behaviour, are possible. For instance, taking into consideration early- and late-season effects which definitely may disturb our findings.

As discussed in Section 2, the point score system of chess ($1,\frac{1}{2},0$) satisfies Equation (3). It might be tempting to speculate on differences between chess and football people based on the choice of the point score system. However, it might be good reason to be careful. As discussed in [19], tests as well as discussions on introducing the 3-1-0 system have been done in chess, maybe unsurprisingly, with the number of drawn matches in chess in mind. However, so far chess has stuck to their incentive-correct system. Still, chess might be an interesting candidate for further empirical analysis regarding collusive behaviour based on game theoretic models, especially, of course, as it contains actual matches between two players, and our model is based on two-player games.

An interesting hybrid, the 3-2-1-0 system, is applied in several hockey leagues—for instance in the Norwegian Get League. Here, 3 points are awarded for a win, 0 points for a loss in regular time. A win in overtime (OT), or on penalties, if OT is time constrained, gives 2 points, while a loss in OT gives 1 point. Suppose the teams at hand are exactly equally good. Then, a win or loss in OT occurs with exactly a probability of a half, and the expected OT-score is 1, 5 ($\frac{1}{2}\cdot 2+\frac{1}{2}\cdot 1$). Hence, given such an assumption, this system can be viewed as a 3-1,5-0 system, or a system obeying Equation (3).

With the (seemingly) increasing problems sport has had with doping, corruption and match fixing, one could ask if it was a good idea to introduce a point score system “begging” for match-fixing. Why not revert to the original 2-1-0, or any point score system one would prefer as long as ω=2δ. It is a simple and cheap transformation, merely including some simple updates of table database software.

## Figures and Tables

**Table 1:** Estimated uncertainty of outcome $\left(\rho_{L}^{j}\right)$, and draw ratio $\left(\delta_{j}\right)$ for some European leagues—2017.

2017	$\rho_{L}^{j}$	$\delta_{j}$
ALBANIA	28.0%	32.8%
AUSTRIA	34.2%	18.9%
BELARUS	15.1%	25.8%
BOSNIA & HERC.	19.5%	28.8%
CROATIA	10.1%	23.9%
CZECH REP.	25.2%	28.8%
ENGLAND	21.0%	22.1%
FINLAND	30.3%	27.8%
FRANCE	27.9%	25.0%
GERMANY	30.9%	24.2%
GREECE	21.4%	27.5%
HOLLAND	24.8%	23.9%
HUNGARY	40.3%	27.3%
ICELAND	35.1%	27.3%
IRELAND	25.9%	23.2%
ITALY	16.4%	22.2%
LUXEMBOURG	26.8%	19.8%
MACEDONIA	25.6%	31.7%
NORWAY	38.8%	24.6%
PORTUGAL	23.6%	26.1%
SPAIN	16.4%	23.4%
SWEDEN	28.0%	28.8%
SLOVENIA	29.6%	30.6%
SWITZERLAND	28.9%	21.1%
TURKEY	26.6%	21.9%

**Table 2:** Regression with draw ratio (δ) as dependent variable and uncertainty of outcome $\left(\rho_{L}\right)$ as independent variable.

Coefficient	Estimate	*t*-Value
Constant	24.35107 ***	8.653
$\rho_{L}$	0.04416	0.423

* *p* < 0.10; ** *p* < 0.05; *** *p* < 0.001.

## Appendix A. Alternative Derivation of the ω = 2δ Result

Suppose two football teams ($T_{1},T_{2}$) are engaged in a match. This match is described through a set of probabilities contingent on strategic combinations made by the two teams. The match has three outcomes; A victory for $T_{1}$ with a probability $p_{12}$, a victory for $T_{2}$ with a probability of $p_{21}$, and a draw probability; $p_{D}=1-\left(p_{12}+p_{21}\right)$.

Although, as defined above, all these probabilities are dependent on team strategies; i.e., $\left(p_{12}|x,y\right)$ where x,y define all possible strategic combinations for the two players, we use the simplified notation without conditionality. Things become unnecessary complicated, and generality is preserved without the full notation.

With these assumptions, and an added assumption of maximal expected point score as team objectives, as well as an assumption of existence of a general point score system (ω,δ,0) (ω points for a win, δ points for a draw and zero points for a loss should provide necessary point scorer system generality); ω>δ>0, $T_{1}$’s expected Pay-off (point score) can be calculated as:

(A1) $$E\left[T_{1}\right]=\omega \cdot p_{12}+\delta \cdot p_{D}$$

Similarly, the expected Pay-off for $T_{2}$ is:

(A2) $$E\left[T_{2}\right]=\omega \cdot p_{21}+\delta \cdot p_{D}$$

Now, as $p_{21}=1-\left(p_{12}+p_{D}\right)$, Equation (A2) can be rewritten as:

(A3) $$E\left[T_{2}\right]=\omega \left[1-\left(p_{12}+p_{D}\right)\right]+\delta \cdot p_{D}$$

What is of interest here, are cooperative incentives. Such cooperative incentives arise, if the combined pay-off (i.e., $E\left[T_{1}\right]+E\left[T_{2}\right]$) are larger for certain strategic combinations different from Nash equilibrium strategies. As a consequence, it seems reasonable to calculate the expression for total pay-off given a certain strategic combination. we find:

(A4) $$E\left[T_{1}\right]+E\left[T_{2}\right]=\omega \cdot p_{21}+\delta \cdot p_{D}+\omega \left[1-\left(p_{12}+p_{D}\right)\right]+\delta \cdot p_{D}$$

Equation (A4) can, after some straightforward algebra be rewritten as:

(A5) $$E\left[T_{1}\right]+E\left[T_{2}\right]=\omega \left(1-p_{D}\right)+2\delta p_{D}$$

Now, the result of interest is evident. Suppose ω=2δ. Then, Equation (A5) collapses to:

(A6) $$E\left[T_{1}\right]+E\left[T_{2}\right]=2\delta \left(1-p_{D}\right)+2\delta p_{D}=2\delta -2\delta p_{d}+2\delta p_{D}=2\delta =\omega$$

That is, the combined Pay-off for any strategy combination is independent of outcome probabilities. This means that as long as ω=2δ, no combined pay-off option exists which are different from any other, and there are no possible incentives for cooperation. Any other configuration (ω≠2δ) provides opportunities for such.

For instance, the present point score system in chess; $\left(1,\frac{1}{2},0\right)$ is OK, as $\left(1=\frac{1}{2}\cdot 2\right)$. The present point score system in football (3-1-0) is **not** OK, as 3≠2·1. However, the former point score system in football – (2-1-0) is OK as 2=2·1.

## Appendix B. Empirical Analysis, Definitions and Results

### Appendix B.1. Definitions

Let us first define some parameters:

$\delta_{ij}$: Number of drawn matches played by team *i* in league *j*
$N_{j}$: Number of teams playing in league *j*
$\rho_{L}^{j}$: Calculated measure for uncertainty of outcome in league *j*
$AP_{ij}$: Actual point score for team *i* in league *j*
$LCP_{ij}$: Least competitive point score for team *i* in league *j*
$MCP_{J}$: Maximally competitive point score for team *i* in league *j*

### Appendix B.2. Calculations

Given knowledge of (Available in most internet table formats) $\delta_{ij}$, it is straightforward to realize that:

(A7) $$\mathrm{The}\;\mathrm{number}\;\mathrm{of}\;\mathrm{drawn}\;\mathrm{matches}\;\mathrm{in}\;\mathrm{league}\;j=\frac{1}{2}\sum_{i=1}^{N_{j}}\delta_{ij}$$

Furthermore, the number of played matches in a double round robin tournament equals the number of matches in each round multiplied by number of rounds. The number of matches played in a round is $\frac{N_{j}}{2}$, while the number of rounds equals $2(N_{j}-1)$. Consequently;

(A8) $$\mathrm{The}\;\mathrm{number}\;\mathrm{of}\;\mathrm{drawn}\;\mathrm{matches}\;\mathrm{in}\;\mathrm{league}\;j=2\left(N_{j}-1\right)\frac{N_{j}}{2}=N_{j}\left(N_{j}-1\right)$$

Then, the ratio of drawn matches in league *j* can be calculated from:

(A9) $$\delta_{j}=\frac{\sum_{i=1}^{N_{j}}\delta_{ij}}{2\cdot N_{j}\left(N_{j}-1\right)}$$

We chose the method used in [10] for our uncertainty of outcome measure. Then, given the notation used here, $\rho_{L}$ can be calculated as:

(A10) $$\rho_{L}^{j}=100\cdot \frac{\sum_{i=1}^{N_{j}}{\left(LCP_{ij}-AP_{ij}\right)}^{2}}{\sum_{i=1}^{N_{j}}{\left(LCP_{ij}-MCP_{j}\right)}^{2}}$$

### Appendix B.3. Data

Results for most top leagues in Europe are shown in Table 1 for the 2017 season (We have used www.altomfotball.no as our primary data source).

In principle, we have chosen all relevant European top leagues as the sample. It seemed as a natural choice due to the continent’s economic as well as performance-wise significance. Some countries are missing due to existence of play-off mechanisms, which makes estimation of both uncertainty and outcome as well as draw ratios highly unreliable for our analysis.

Simply by observing Table 1, we observe (significant) differences in estimated uncertainty of outcome data ($\rho_{L}^{j}$), while not obvious (significant) differences in draw ratios ($\delta_{j}$).

### Appendix B.4. Empirical Analysis

Based on the data in Table 1, the simple linear regression:

(A11) $$\delta_{i}=\beta_{0}+\beta_{1}\cdot \rho_{L}^{i}+\epsilon_{i}$$

is estimated. The results (using R (https://www.r-project.org)) are shown in Figure 3 and Table 2.

As the graphical image in Figure 3 as well as the actual *t*-test on the regression coefficient, $\beta_{1}$ in Table 2 indicates, the null hypothesis cannot be rejected, and no association between draw ratio and uncertainty of outcome is identified.
